# Recent and Projected Increases in Atmospheric CO_2_ Concentration Can Enhance Gene Flow between Wild and Genetically Altered Rice (*Oryza sativa*)

**DOI:** 10.1371/journal.pone.0037522

**Published:** 2012-05-23

**Authors:** Lewis H. Ziska, David R. Gealy, Martha B. Tomecek, Aaron K. Jackson, Howard L. Black

**Affiliations:** 1 Crop Systems and Global Change Laboratory, United States Department of Agriculture, Agricultural Research Service, Beltsville, Maryland, United States of America; 2 Dale Bumpers National Rice Research Center, United States Department of Agriculture, Agricultural Research Service, Stuttgart, Arkansas, United States of America; University College London, United Kingdom

## Abstract

Although recent and projected increases in atmospheric carbon dioxide can alter plant phenological development, these changes have not been quantified in terms of floral outcrossing rates or gene transfer. Could differential phenological development in response to rising CO_2_ between genetically modified crops and wild, weedy relatives increase the spread of novel genes, potentially altering evolutionary fitness? Here we show that increasing CO_2_ from an early 20^th^ century concentration (300 µmol mol^−1^) to current (400 µmol mol^−1^) and projected, mid-21^st^ century (600 µmol mol^−1^) values, enhanced the flow of genes from wild, weedy rice to the genetically altered, herbicide resistant, cultivated population, with outcrossing increasing from 0.22% to 0.71% from 300 to 600 µmol mol^−1^. The increase in outcrossing and gene transfer was associated with differential increases in plant height, as well as greater tiller and panicle production in the wild, relative to the cultivated population. In addition, increasing CO_2_ also resulted in a greater synchronicity in flowering times between the two populations. The observed changes reported here resulted in a subsequent increase in rice dedomestication and a greater number of weedy, herbicide-resistant hybrid progeny. Overall, these data suggest that differential phenological responses to rising atmospheric CO_2_ could result in enhanced flow of novel genes and greater success of feral plant species in agroecosystems.

## Introduction

Gene transfer between organisms is acknowledged as a significant influence on evolutionary change [1]. Consequently, a scientific understanding of biotic and abiotic factors that promote gene flow is of considerable interest. Some biotic conditions are already well-recognized. For example, in plant populations cross pollination must occur with synchronicity in flowering, spatial proximity and genetic compatibility [2]. Naturally occurring abiotic or physical phenomenon are also understood; for example, the role of wind in pollen transfer for anemophilous plants, such as ragweed.

However, in addition to these natural drivers, there is increasing recognition that gene flow can be altered by anthropogenic activities [3], [4]. One prominent feature of anthropogenic disturbance that has been well quantified globally is the increase in atmospheric carbon dioxide, CO_2_. Since 1959, concentrations of atmospheric CO_2_ have increased from 318 to 392 µmol mol^−1^; and, depending on anthropogenic emission rates, may exceed 1000 µmol mol^−1^ by the end of the century [5].

Carbon dioxide is highly relevant to biology as it represents the sole source of carbon available in plant metabolism. Increases in CO_2_ concentration *per se* have been shown in numerous studies to increase carbon fixation with subsequent effects on plant growth, development, morphology and reproduction [6]. It is also evident that inter and intraspecific variation to rising CO_2_ occurs [7], [8], and such variation could lead to differences in reproductive phenology and gene flow. These latter differences have never been quantified. Their occurrence, however, given the inclusive nature of the CO_2_ increase, has extensive biological consequences since it suggests a specific human-disturbance capable of altering gene transfer for plant communities globally.

One plant community where gene flow is of explicit interest is the agro-ecosystem. The introduction of novel genes in domesticated plant taxa has led to concerns that gene flow between genetically transformed and uncultivated relatives could alter the fecundity and management of feral plant species [9] with subsequent impacts on their evolutionary fitness [2], [10], [11]. For agro-ecosystems, increased gene exchange either from the crop to the related wild/weedy species–or vice-versa–is consequential, since the end result will be an introduction of non desired traits (e.g. herbicide resistance) and the formation of an unwanted biotype of the cultivated crop.

Historically, wild or weedy rice (henceforth referred to as wild rice) has been controlled through utilization of a transplant flooded culture system. With modern advances in mechanization and selective herbicides, and declining water availability globally, direct seeding systems have been increasingly emphasized. As this trend has continued, wild rice has emerged as a pervasive and serious impediment to the productivity of cultivated rice globally [12], [13]. Often called red rice due to the coloration of its pericarp, wild rice is the same species as the cultivated crop (*Oryza sativa*; not to be confused with *Oryza rufipogon* or *Zizania latifolia* Griseb.). It can be very difficult to control, because seasonal shattering allows seed bank retention, while undispersed weed seeds may be inadvertently collected and replanted in subsequent crops.

Wild and cultivated rice are primarily self-pollinated [14]. However, each type can outcross at significant levels depending on the environment and their genetic background [15], [16]. Overall, rice is recognized globally as one of the five most-widely grown crops prone to gene flow [17].

An emerging strategy to manage wild rice is to apply herbicides in conjunction with the implementation of herbicide-resistant cultivated rice. One such herbicide resistant rice cultivar is “Clearfield™ 161” (CL 161). Derived from chemical mutagenesis (non-transgenic), this and related cultivars are resistant to acetolactate synthase-inhibiting imidazolinone herbicides such as imazethapyr [14], and are now widely grown in the southern U.S. where wild rice infestation occurs. However, if potential transfer of herbicide resistance between wild and cultivated rice populations were to occur in response to rising CO_2_, it would accelerate the introduction of herbicide resistant hybrids into rice systems with subsequent effects on evolutionary fitness, diversity, spread and management of wild rice.

For this study we apply the hypothesis that recent 20th century CO_2_ increases (300 to 400 µmol mol^−1^), and those projected for the 21 century (up to 600 µmol mol^−1^), could differentially affect the spatial and temporal development of reproductive traits in wild and cultivated rice with modifications in phenological development, outcrossing rates and gene transfer between populations. The data presented here confirm this hypothesis and indicate that rising CO_2_ may be an additional factor in the spread of novel genes between wild and cultivated rice populations.

## Results

**Figure 1:**
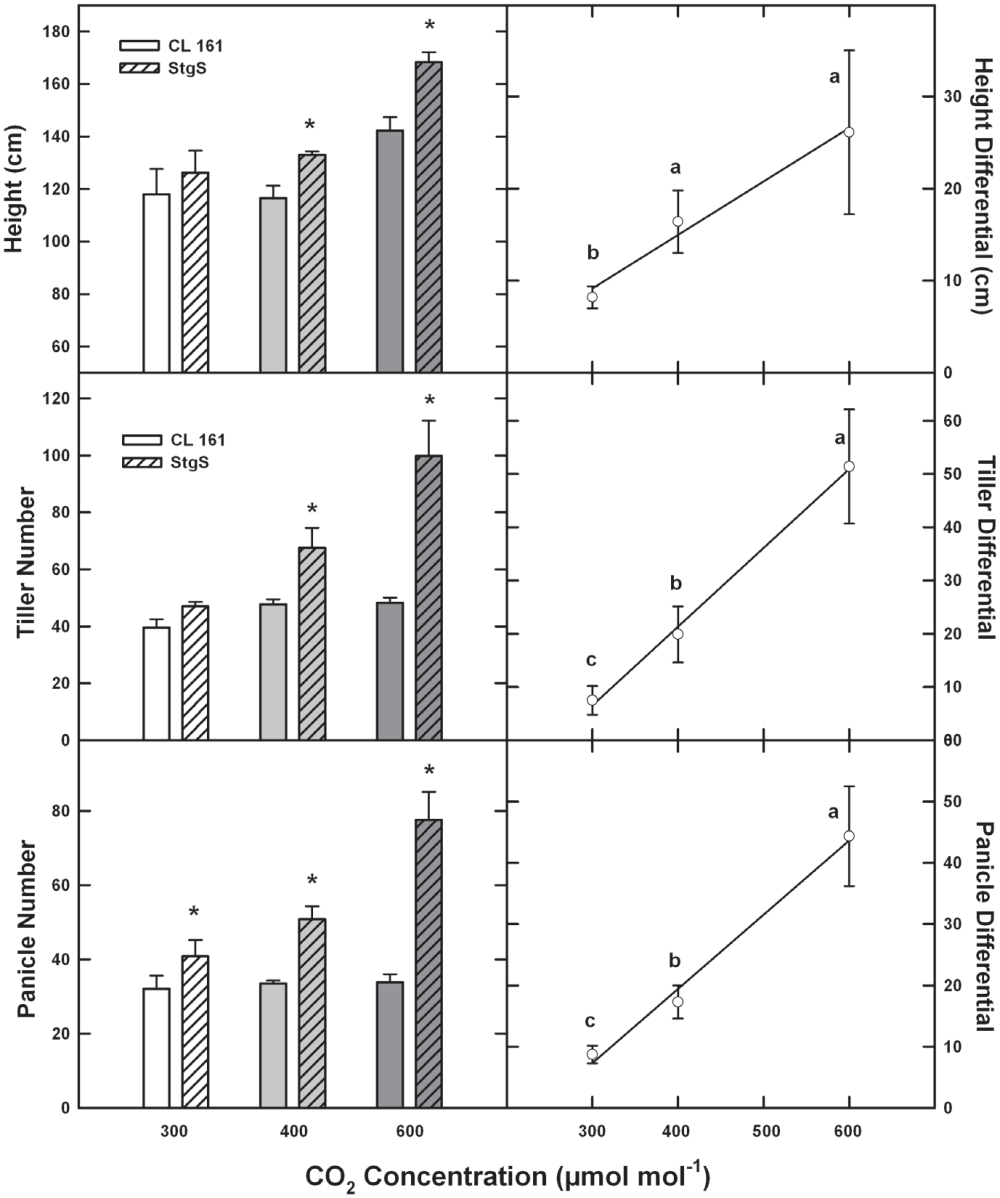
Relative change in morphological parameters (per plant) that influence reproductive onset and outcrossing as a function of recent and projected increases in CO_2_ concentration (µmol mol^−1^) for a wild and cultivated rice population. * indicates a significant difference at the P<0.05 level as a function of a given CO_2_ concentration between populations; different letters indicate a significant difference in the degree of CO_2_ enhancement for the measured parameter (e.g. height). Bars are ±SE.

Specific developmental patterns of interest that would be associated mechanistically with directional gene flow from reproductive structures would include changes in height at anthesis, changes in tiller and panicle formation, and synchronicity of flowering times between populations. An examination of these parameters included a test of both populations at planting frequencies associated with cultivation; i.e. approximately 2 wild rice plants per m^2^ of cultivated rice, utilizing environmental chambers at constant macro-environmental conditions in order to maintain consistent CO_2_ values between early 20^th^ century and mid-21^st^ century levels. It is clear that as CO_2_ increased, plant height, tiller production and panicle number all increased to a greater extent in the wild vs. the cultivated populations (Figure 1). Plant height is thought to be an important determinant of pollen outcrossing and gene flow, given the limited range of pollen release in rice [14]; here CO_2_-induced height differences between the cultivated and wild populations increased from 8 to 26 cm (Figure 1). Tiller and panicle numbers, which provide the structural basis for floral initiation, also rose to a greater extent for the wild relative to the cultivated populations doubling in response to the CO_2_ increase from 300 to 600 µmol mol^−1^ (Figure 1).

**Figure 2:**
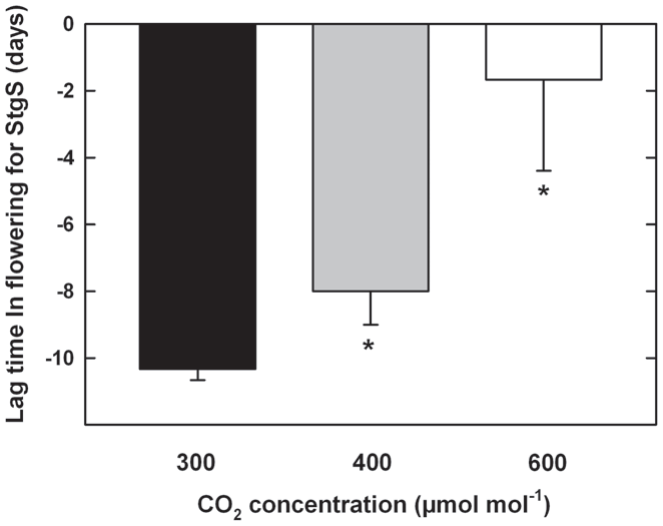
Lag time in days following flowering in the cultivated rice population (CL 161) that wild rice (StgS) flowered as a function of CO_2_ concentration. * indicates a significant difference relative to the 300 µmol mol^−1^ baseline; Bars are±SE.

In rice, gene transfer escalates when flowering times (anthesis) are in sync between wild and cultivated populations. Interestingly, anthesis for the cultivated population was not affected, whereas in contrast, flowering times were initiated earlier (∼8 days) in response to rising CO_2_ for the wild population (Figure 2).

**Figure 3:**
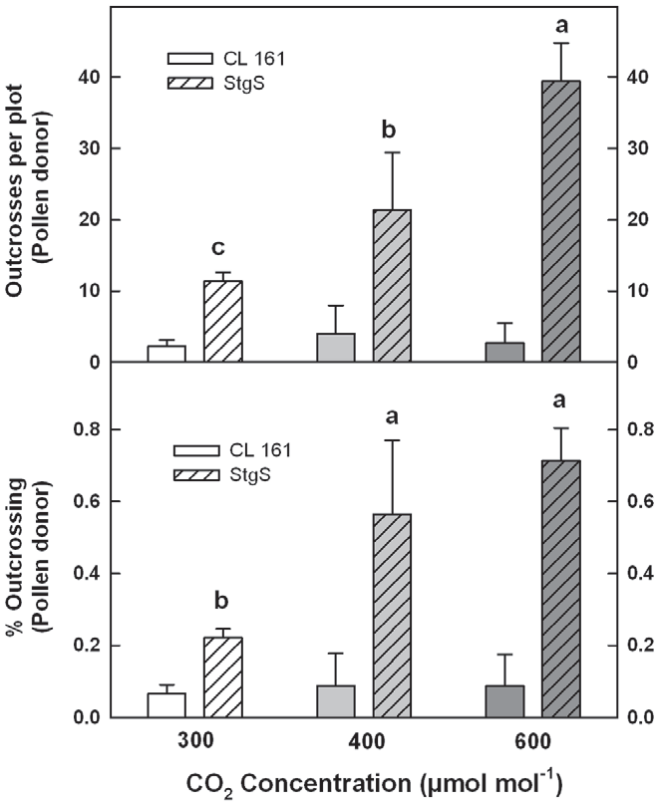
Percent outcrossing and outcrosses per plot for the pollen donor as detected in cultivated and wild rice populations in the field plots. Outcrosses were always significantly higher for the StgS relative to the CL 161 population regardless of CO_2_ concentrations (not shown). Different letters indicate a significant population by CO_2_ concentration interaction for lthe StgS relative to the CL 161 population. Bars are ±SE

Field evaluation of seed collected from these populations indicated that outcrossing from the cultivated to the wild population was low, and did not change as a function of CO_2_. In contrast, outcrosses per plot, and the percent outcrossing significantly increased from the wild population relative to the cultivated line as a function of CO_2_ concentration (Figure 3). This disproportionate increase in outcrossing and gene transfer from the wild population was associated with the morphological and phenological changes arising from the increase in atmospheric CO_2_, namely, increased height, greater floral (panicle) production and enhanced temporal initiation of anthesis.

## Discussion

Biotic or abiotic factors that differentially impact outcrossing and gene flow between species will influence population size, genetic diversity, metapopulation dynamics and, potentially, the rate and trajectory of evolution [18].

There are compelling arguments that human activity, from habitat destruction to global warming, is beginning to change reproductive biology and outcrossing in plant communities (3). To date, much of this work is focused on how anthropogenic disturbances are likely to change species range, demography [19], pollinator populations [20], and plant phenology [21], with little empirical evidence regarding outcrossing rates and gene transfer.

Among anthropogenic factors, CO_2_ has a fundamental influence on plant metabolism, independent of any effect on climate. For example, with respect to reproduction, increasing CO_2_ has been shown to alter flowering times [22], increase pollen amounts [23], increase flower numbers and size [24] and enhance nectar production [25]. Differential reproductive responses between and within plant species have also been widely documented in response to atmospheric CO_2_ increases above pre-industrial concentrations [26]. However, a clear association between differential species responses to CO_2_ and quantification of gene flow has been unavailable.

Here the disparate responses to recent and projected CO_2_ between related rice lines resulted in a directional increase in gene flow from feral to cultivated populations. Such gene shifts have been noted previously in rice systems [14], emphasizing that gene flow can occur in both directions. This may seem counter-intuitive given the relative population ratios of cultivated to wild rice (i.e., ca 7∶1); however increases in height, tiller and panicle production, and synchronicity of flowering observed here for wild rice are consistent with facilitating gene flow from wild to cultivated rice plants [27].

We emphasize that for agro-ecosystems, increased gene flow in either direction is of consequence since the trait of interest (whether herbicide resistance or any other gene introduced via mutation or genetic engineering) will now be associated with an undesirable biotype of the preferred crop. That is, Clearfield plants that receive wild rice genes will produce hybrid seed whose phenotypic characteristics, including herbicide resistance, shattering, nutritional composition, etc., are undesirable relative to the quantitative and qualitative traits of the cultivated rice [27].

Therefore, the CO_2_ induced changes in gene flow observed here enhanced the creation, occurrence and perpetuation of ‘weedy’, herbicide-resistant hybrid progeny. Such progeny, in turn, will have negative consequences with respect to rice yields (e.g., enhanced competition), quality (e.g. nutrition) and weed management (e.g. herbicide resistance).

The results reported here for the differential response to rising CO_2_ are consistent with previous results for multiple red rice biotypes vs. cultivated lines when grown at intervals of 100 µmol mol^−1^ [28] above the pre-industrial CO_2_ baseline. It has been suggested that the greater degree of genetic and phenotypic plasticity among wild or weedy rice biotypes may allow for greater exploitation of abiotic resources (e.g., CO_2_) as compared to commercial rice cultivars that have been developed to be phenotypically uniform, early in maturity, and short in stature [28], [29].

Because of selection pressures associated with weed management, many of the plant species present in agroecosystems are genetically related to the desired crop species [30]; consequently it is recognized that outcrossing can, and does occur (e.g., oat and wild oat, sorghum and shattercane, nightshade/bittersweet and potato, cultivated and wild sunflower *inter alia*) [31], [32], [33], [34]. Based on the results shown here for rice, studies on outcrossing and gene flow should also be conducted to ascertain whether the ongoing increase in atmospheric CO_2_ has, or will, increase the spread of novel genes via hybridization for these other agroecosystems. The greater introduction and spread of such genes (e.g. herbicide resistance) will have significant effects on pest management and food security.

## Materials and Methods

Controlled environment chambers (EGC Corp., Chagrin Falls, OH) were used because, at present, no methodology is available that can expose plants to sub-ambient carbon dioxide concentrations [CO_2_], for 24 h day^−1^ [35]. Temperature for each chamber was varied in a diurnal mode from an overnight low of 22°C, to a maximum afternoon value of 32°C, with an average daily (24 h) value of 24.4°C. Light as photosynthetically active radiation (PAR) varied diurnally in conjunction with temperature, with the highest PAR (∼900 µmol m^2^ s^−1^) occurring during the afternoon. The daily light period was 14 h, supplied by a mixture of high-pressure sodium and metal halide lamps, and averaged 20.9 mol m^−2^ day^−1^ for all chambers. Injection of either CO_2_ or CO_2_-free air was determined using a TC-2 controller that monitored [CO_2_] measured from an absolute infrared gas analyzer (WMA-2, PP Systems, Haverhill, MA, USA). Carbon dioxide concentrations were set at 300, 400 and 600 µmol mol^−1^ 24 h day^−1^. These concentrations approximated the carbon dioxide values present during the end of the 19^th^ century, the current value, and that projected by the end of the 21^st^ century [5]. Mean 24 h day^−1^ [CO_2_] values (±SD) throughout the experiment were 312±17.3, 413±18.6, and 624±31.5 µmol mol^−1^. Temperature, humidity and [CO_2_] were recorded every 15 min and averages determined on a daily basis for all experimental runs.

An awnless, strawhull red rice type from Arkansas, StgS (PI653423; henceforth referred to as StgS wild rice), typical of the most widespread biotypes in Arkansas/southern U.S.A. [36], was selected as the outcrossing partner for cultivated rice. Bulk seed were obtained from a collection maintained by USDA-ARS. CL 161 rice, which contains resistance to imazethapyr herbicide (HR) was selected as the outcrossing partner for wild rice. Seed are available from a commercial source (Horizon-Ag, Memphis, TN). Seed of these *O. sativa* biotypes were planted at commercial densities: CL 161 rice at ∼25 plants per m^2^ and StgS at 2 plants m^2^ in each of three chambers, with each chamber programmed to one of the CO_2_ treatment concentration. All plants were watered to the drip point daily with a complete nutrient solution. Non destructive observations of phenology, including emergence, tillering, height, heading and flowering were determined for each biotype and CO_2_ treatment. Plants were grown until maturity (final harvest) which was determined when >50% of the panicles for both biotypes had senesced. Because seed from wild rice shatters, three panicle subsamples from individual StgS plants for each experimental treatment were wrapped in cheesecloth, and an allometric relationship between seed and panicle weight was used to estimate seed yield. At maturity individual plants were cut at soil level, tillers and panicles counted before separation into tillers, panicles, seed and leaf laminae. Because of the difficulty in separating roots of cultivated and wild rice, no attempt was made to determine root biomass. Seed yield was determined for CL 161 rice by gently shaking panicles following cutting. All plant material was dried at 65°C or until dry weight was constant. Seeds were separated by biotype, air dried and corrected to 10% moisture.

At the end of a given experiment, the CO_2_ treatments were randomly reassigned, and the entire experiment repeated with runs over time considered as replications (blocks). This occurred over a two year period from 2009 through 2010, with three runs for the 300 and 600 µmol mol^−1^ and two runs for the 400 µmol mol^−1^ CO_2_ concentrations. PAR, humidity and temperature were quantified prior to, and at the end of each run in order to determine within chamber and among chamber variability. Values for temperature, PAR and humidity were consistent among chambers and runs. All measured and calculated growth and reproductive parameters were analyzed using analyses of variance including CO_2_ concentration, run and biotype (StgS vs. CL 161) as fixed effects (Statview software, Cary, NC, USA). Seed from each run and biotype was collected and shipped to the USDA-ARS Dale Bumpers Rice National Rice Research Center in Stuttgart, Arkansas.

Before planting, the weights of two groups of 100 randomly selected seeds of both rice and StgS from each growth chamber harvest were averaged so that a constant number of seeds could be weighed out and planted in each screening plot. Seeds harvested from the growth chambers in August 2009 to February 2010, and in December 2010 to May 2011 were planted in the field on May 5, 2010, and May 9, 2011, respectively, at the University of Arkansas Rice Research and Extension Center located near Stuttgart, AR (34.49° N, 91.55° W). Soil was a DeWitt silt loam (fine smectitic, thermic, Typic Albaqualfs) with 1.2% organic matter and pH 5.8. Cultivated and wild rice were drill-seeded at a depth of approximately 2 cm into separate plots 12.2-m-long with nine rows spaced 18 cm apart. Seeding density was 370 seeds m^−2^. This was equivalent to 7200 seeds per 12.2 m×1.62 m plot. Plots were firmed with a roller to facilitate seed-soil contact and assist seed germination.

Herbicides were applied to plots to control barnyardgrass and other weeds as necessary. Clomazone + quinclorac (0.26 kg active ingredient (ai) ha^−1^+0.21 kg ai ha^−1^) were applied after planting (pre-emergence to late pre-emergence). In order to ensure complete kill of all non herbicide-resistant wild rice plants (i.e. plants that did not contain the HR gene), imazethapyr + CS-7 surfactant (0.07 kg ai ha^−1^+0.25% v/v; recommended rate), was applied 15 to 18 days after crop emergence when rice was at the 3 to 4 leaf stage and 15 cm tall, and again at 1 and 2 weeks after the initial application. Surviving plants in plots planted with StgS rice seeds were considered to be putative F_1_ hybrids that had acquired the HR gene from CL 161 pollen [15], [16] as a result of natural outcrossing in the growth chambers. In plots planted with CL 161 seeds, tall, late-flowering plants with pubescent leaves were considered to be putative F_1_ hybrids [27] that had acquired StgS genes from natural outcrossing in the growth chambers. Urea at 34 kg nitrogen ha^−1^ was broadcast over all plots before establishment of the permanent flood on June 18, 2010, and June 27, 2011.

Healthy, green leaf tissue sampled from putative F_1_ hybrid plants in field plots were collected in September of each year and kept frozen until DNA extraction. DNA was extracted and polymerase chain reaction (PCR) was performed following procedures described previously [37], [38]. Samples were separated on an ABI Prism 3730 DNA Analyzer (Applied Biosystems, Foster City, CA) and the sizes of SSR fragments were determined and alleles binned using GeneMapper version 3.7 software (Applied Biosystems, Foster City, CA).

Six genetic markers (Rid12, RM5, RM232, RM234, and RM253, and RM 277) previously shown to differentiate between CL 161 rice and StgS or closely related wild rice types [36], [39] were selected to determine the genotypes of putative hybrid plants. True F_1_ hybrids between CL 161 and StgS were confirmed as being heterozygous for the six selected loci. In true hybrid plants, the expected allele size pairings (rice/wild rice) for genetic markers were: Rid 12 (151 bp/165 bp), RM232 (156 or 154 bp/148 bp), RM234 (135 bp/158 or 154 bp), RM253 (131 bp/139 or 141 bp), and RM 277 (114 bp/118 bp). Outcrossing rates were calculated as: 100×(number of true F_1_ hybrids in screening plot)/(number of seedlings in screening plot). Data on outcrossing rates and gene flow were tabulated for each year, biotype and CO_2_ treatment for all field plots. Data were analysed using analyses of variance with chamber run, CO_2_ concentration, year, plot and biotype (StgS vs. CL 161) as fixed effects (Statview software, Cary, NC, USA).
